# Influence of Emotionally Charged Information on Category-Based Induction

**DOI:** 10.1371/journal.pone.0054286

**Published:** 2013-01-23

**Authors:** Jennifer Zhu, Gregory L. Murphy

**Affiliations:** Department of Psychology, New York University, New York, New York, United States of America; University of Milan, Italy

## Abstract

Categories help us make predictions, or inductions, about new objects. However, we cannot always be certain that a novel object belongs to the category we are using to make predictions. In such cases, people should use multiple categories to make inductions. Past research finds that people often use only the most likely category to make inductions, even if it is not certain. In two experiments, subjects read stories and answered questions about items whose categorization was uncertain. In Experiment 1, the less likely category was either emotionally neutral or *dangerous* (emotionally charged or likely to pose a threat). Subjects used multiple categories in induction when one of the categories was dangerous but not when they were all neutral. In Experiment 2, the most likely category was dangerous. Here, people used multiple categories, but there was also an effect of avoidance, in which people denied that dangerous categories were the most likely. The attention-grabbing power of dangerous categories may be balanced by a higher-level strategy to reject them.

## Introduction

Categories help us organize and apply information about the world around us. When we need something to write with, we pick up a pen because we know that pens are writing utensils. Even though we may not have seen that particular pen before, we can infer its function via knowledge of its category. Thus, one especially important function of categories is that they allow us to make *category-based inductions*, or *predictions* about novel items.

When making a category-based induction, you cannot always be certain what category a novel item belongs to. A half-covered object on your desk might be a pen or a pencil. If you're just looking to jot down a note, it doesn't make any difference which it is, because either one will serve this purpose. The uncertainty in categorization doesn't change your induction about this object (you can write a note with it). If you're looking to sign a legal document, however, then the pencil won't be helpful, and now the uncertainty over categorization should create an uncertainty over the subsequent induction (can you sign with it?). This example illustrates the principle that in order to make accurate predictions about an object, you should take into account the different categories it might be in and the properties associated with those categories [1], [2]. The present research continues an investigation of whether and when people do this when making inductions.

In one series of experiments, we and our collaborators have provided subjects (students at an American university) with visual displays of categories, so that the exact probabilities of classification and feature prediction can be experimentally controlled [3], [4], [5]. Subjects answered induction problems about objects that are most likely (about 65%) to be in a *target* category but somewhat likely (about 35%) to be in an *alternative* category. In these studies, we have generally found that only about 25% of people [3] take into account both categories when making a prediction. The majority of responses focus only on the target category, as if it were certain to be correct, leading to suboptimal predictions.

Another series of studies used a paradigm in which people read stories and made predictions about characters or items in the story whose categorization was uncertain. In this paradigm (used in the present experiments), it is not possible to identify individual subjects as basing their inductions on a single or multiple categories. However, group responses consistently show little or no evidence that the alternative categories are used in making inductions. Again, people tend to focus on the target category, as if it were certain [6], [7].

Consider an example from one of those earlier studies [7]. In this scenario, Betty had landed in the hospital after breaking her hip. Considerable exposition explained her situation, mentioning that her eyesight was not very good. She sees a figure in white coming towards her room, whom she believes is the nurse. However, she also realizes that it might be the orderly, who was described earlier in the scenario. Subjects are asked to make predictions about this figure in white, such as whether the person would be likely to get Betty clean sheets if asked and whether the person would answer a question about her medication. If people believed that the figure in white was most likely a nurse but possibly the orderly, then the effect of the orderly would be to raise the prediction about the sheets but lower the prediction about the medication. Other subjects read a similar story in which the figure in white was probably the nurse but possibly Betty's doctor. Now the alternative category, doctor, would lower the prediction about sheets but raise the prediction about answering the question—the opposite of the effect of the orderly. By comparing these predictions across subjects who received different alternative categories, we could evaluate whether the categories had the expected effects. In fact, under most conditions, people's answers to these predictions do not differ across these two groups [6], [7], [8]. That is, once people decide that the person in white is most likely the nurse, it makes no difference whether the person might instead be an orderly or doctor, even though the probability of the alternative category ranged from 25–50% (as rated by other subjects). It seems that only the target category is used in making predictions, even when it is uncertain.

We have described this effect as an example of Evans's [9] *singularity principle*, that people tend to focus on only one situation or possibility, unless forced to do otherwise. It is also related to Stanovich's [10] characterization of reasoners as *cognitive misers*, who often accept answers that come to mind easily, without feeling the need to check their work. A brief summary of our explanation is that when people identify a most likely category, they rely on it in making predictions unless some external factor also draws the alternative category into working memory (see [5] for discussion). Indeed, even in the limiting case in which two categories are equally certain, most subjects simply guess which one might be correct and use it for their induction [3], [11].

In presenting this research, we have occasionally been asked what would happen if the alternative category were particularly attention-grabbing or worrisome. For example, if a hidden animal was probably a kitten but possibly a rattlesnake, would people really ignore the rattlesnake possibility? That seems unlikely, though it is also possible that people would simply shift to a new target category, acting as if the animal is a rattlesnake and ignoring the kitten possibility. This would be an influence of the rattlesnake category, but it would not represent improved reasoning by the use of multiple categories. Our original materials [6] did have an example of this type, in which someone walking up the driveway might have been a real estate agent or instead a burglar. However, the majority of alternative categories were not of this sort, and the experiment was not designed to test the influence of such categories.

It would make good sense for people to attend to what we will call *dangerous* categories—those that evoke negative reactions (like bats or rats) or pose a threat (like weapons or serious diseases). Considerable research suggests that some negative stimuli attract attention automatically (e.g., [12], [13]). As is well known, losses outweigh gains in their effect on decisions [14], and negatively valenced stimuli evoke larger ERP responses during evaluations than positive ones do [15].

Such considerations suggest that in category-based induction, if an alternative category is negative, it may remain active in working memory even when people decide another category is more likely. That is, the negative valence could actually improve reasoning, by encouraging people to consider both possibilities when making their predictions. Evidence for this possibility has been found by Hayes and Newell [16]. In their study, subjects diagnosed a person with a fictional disease and then predicted what other symptoms would be present. However, the diagnosis was not certain, as two diseases fit the symptoms to varying degrees. In their “response-cost” condition, a message indicating that the alternative (less likely) diagnosis “is very serious and possibly terminal” appeared while people made their predictions. Subjects did use multiple categories in inductions in such a condition. Similar results were found when the alternative was labeled as serious prior to the prediction phase.

These results support the notion that people could use multiple categories when the alternative is negative. However, it would be more convincing to see an effect with natural categories rather than artificial categories explicitly labeled as “very serious and possibly terminal,” which could be subject to demand characteristics. Furthermore, it is possible that with natural dangerous categories, the primary effect will be for people to switch to them as the basis for induction rather than to use multiple categories, as in our rattlesnake example. For example, Gigerenzer [17] argued that the perceived “dread risk” of airplane crashes after September 11, 2001 caused people switch to using other, actually riskier forms of transportation that were not associated to the recent terrorist attacks.

No doubt most inductions involve fairly harmless and unthreatening categories. However, there is no shortage of dangerous categories in the world, and understanding how they influence induction is important, because those inductions may be the most consequential. In medical decisions, even a small possibility that a condition is caused by a serious disease may greatly affect decisions about whether and how to treat the condition. In personal interactions, it may not be clear whether a person you've agreed to meet for a date is going to be pleasant or possibly someone you are desperate to get away from. People invest in stocks that they expect to increase in value, but if there is a low-probability event that would devastate the stock price (e.g., natural disaster or unfavorable legislation), how will that influence potential investors' predictions of its value? They might say, “that probably won't happen” and ignore the low-probability event, or they might overweight the catastrophic possibility.

In short, when a dangerous category is a possibility, it may have an outsized effect on people's predictions about what will happen. Experiment 1 examined this possibility by using scenarios with a neutral (i.e., not dangerous) category that was most likely and an alternative category that was either dangerous or not, depending on condition. In this design it was up to the subjects to note the valence of the alternative and to decide whether to use it as part of a prediction about an object that could be in either the target or alternative category. If dangerous categories intrude on working memory during prediction, then the results should show that they, but not the neutral alternative, influence predictions.

## Experiment 1

### Methods

#### Subjects

Thirty-one New York University undergraduates completed the experiment for class credit. The study was approved by the NYU Institutional Review Board, and subjects in all experiments gave written consent to participate.

#### Materials

***Story construction*** *.* We constructed two versions of 12 stories that mentioned different categories (listed in Table 1). In one story, Brian complained of a sore throat, and the doctor said it was most likely a throat infection (the *target* category). In one version of the story, the doctor added that the pain might instead be due to a cold (*neutral* alternative), and in the other version, the doctor said the pain might instead be due to throat cancer (*dangerous* alternative). All subjects answered the same five questions after reading a story (see Table 2). Questions 1 and 3 were filler questions. Questions 2 and 4 were the feature probability judgments. Question 2 (surgery) was congruent to cancer while question 4 (fever) was congruent to a cold (see pretest below). The surgery question should get a higher rating when cancer was mentioned as a possibility in the story than when cold was mentioned, and vice versa for the fever question—*if* people take the alternative categories into account. Question 5 asked subjects which category they believed was most likely.

**Table 1:** Categories used in Experiment 1.

Target	Dangerous Alternative	Neutral Alternative
dog	rat	kitten
swimmer	shark	school of fish
gummy vitamins	narcotic painkillers	M&Ms
classmate	creepy stalker	guy on whale cruise
town council president	gang member	store manager
swallows	bats	robins
real estate agent	murderer	cable tv worker
throat infection	cancer	cold
cell phone	revolver	instant camera
man's son	kidnap victim	neighborhood boy
brother tickling	snake	wind
meeting about party	meeting about getting suspended	meeting about winning internship

*Note.* The narcotics and snake items were dropped from analysis in Experiment 1.

**Table 2:** Sample Story, Experiment 1.

Brian had a physical exam scheduled with his doctor. When the day arrived, he had been having a sore throat for a couple of days. The doctor looked at it and took a throat swab. She told him, “I'm pretty sure it's just a minor throat infection. I've been seeing a lot of them recently, and yours looks like most of them. But given the amount of pain you're describing, there's also a small possibility that you have [a cold/throat cancer]. But let's see how the throat culture turns out. If it's positive, then I'll prescribe some antibiotics for you.” The rest of the physical went fairly well, except that Brian had put on 7 pounds, and the doctor told him to try to cut some fat out of his diet.
**Questions**
1. What is the probability that Brian will actually cut some fat out of his diet?
2. What is the probability that Brian will need to undergo surgery in the near future?
3. Why did Brian see his doctor?
4. What is the probability that Brian will have a fever in the near future?
5. What do you think Brian's throat pain is **most likely** caused by? (Please give only one answer.)

The materials were divided into two sets such that half the stories in one set contained the dangerous alternative and half the neutral alternative, the assignment being switched in the other set. Thus, each subject only read one version of a story, but the two versions were read equally often across subjects. Question order was varied across stories.

#### Pretesting

To ensure that the alternative categories differentially predicted the two induction features, 20 other subjects filled out questionnaires asking for the probabilities that a given feature would be found given a category. Each question gave a sentence or two to set the context (e.g., “Brian had a physical exam and complained about a sore throat.”) and then provided just one of the three classifications without any uncertainty being expressed (e.g., “The doctor took a throat swab and told him that it was a minor throat infection.”). Following this, subjects rated two or three potential features (e.g., “What is the probability that Brian will have a fever in the near future?”). Three forms were used, varying in which category was mentioned, and subjects completed all forms, in rotated orders.

The results showed that the selected features were congruent to their intended categories. For example, when Brian was told he has throat cancer, subjects rated Brian as having an 88% probability of future surgery and 8% probability of fever. On average, the congruent features were rated 56.3% higher than the incongruent features (55% and 58% for the dangerous and neutral alternatives after omitting items not included in the main analyses below). One alternative category, the flu, was replaced with a cold after pre-testing, because we became aware that some subjects found it (somewhat) dangerous.

To ensure that the dangerous alternative was considered to be something people would want to avoid, we carried out another test in which 19 subjects rated the categories and features in isolation as to how “desirable” they were (following [13]), on a −5 to +5 scale. The instructions explained that −5 would correspond to something that was “extremely undesirable with terrible consequences” and +5 that “the thing is extremely desirable with very positive consequences.” The target and neutral alternatives were mildly positive (*M* = 1.0 and 1.3, respectively). The dangerous categories were rated as undesirable, with a mean of −3.7. All of the dangerous categories received negative ratings except for narcotic painkillers (*M* = .06), which were nonetheless much less positive than their neutral categories M&Ms and gummy vitamins (3.17 and 2.94). (The dangerousness ratings probably underestimated the dangerousness of some items outside of their story contexts. Narcotics are fine in their place but not in the grasp of a hungry toddler, as in our story.)

Furthermore, the features that were congruent to the neutral categories were also somewhat positive (*M* = 1.4), and the features congruent to negative categories were correspondingly negative (*M* = −2.6). This was true of every scenario. In short, the categories and features intended to be dangerous were in fact viewed as things that were undesirable and to be avoided.

#### Procedure

The experiment was conducted with paper and pen. Subjects were given a packet of 12 stories and written instructions. The instructions explained that we were interested in how people understand stories. Some of the questions would be factual and some would be probability-based to be answered on a scale of 0–100%. 0% meant something would never happen while 100% meant something would always happen. Subjects answered questions at their own pace.

### Analysis

The goal of this experiment was to determine whether people would use multiple categories in induction when the alternative was emotionally charged compared to when it was neutral. To determine whether subjects attended to the alternative category as well as the target category, we examined whether they changed their probability ratings to a given question depending on the alternative category. For example, the question, “What is the probability Brian will have a fever” is congruent when the alternative category is the cold and incongruent when the alternative category is cancer (according to subjects' ratings). When the question asked how likely it is that Brian will have a fever, the congruent induction (cold alternative) minus the incongruent induction (cancer alternative) represented how much subjects attended to the second category. If people attend only to the target category, then the difference scores will be about 0, because the target is identical in both conditions.

Inductions are potentially influenced by both target and alternative categories. For example, if a subject responded that it was 75% likely Brian would have a fever when the story mentioned the throat infection (target) and the cold (alternative), this probability rating may be due to both the infection and the cold. In order to distinguish between the effects of the target and alternative categories, the target category pretest score was subtracted for each prediction question. In the pretest story that stated Brian definitely had a throat infection, subjects rated it 49% likely on average that Brian would have a fever. In the test phase story about the throat infection and cold, 49% was subtracted from the 75% response to account for the effect of throat infection. The difference, 26%, provided an estimate of how much the alternative category, the cold, influenced the induction. The same target pretest subtraction was done for every feature prediction question. We report analyses based on the raw scores as well as these corrected scores.

In order to determine whether subjects used multiple categories, we only included trials in which they chose the target category (e.g., throat infection) as most likely. If they thought the alternative category was most likely, this would change their inductions apart from any use of the alternative category. That is, the predictions only hold when people think the target category is most likely, so trials on which subjects did not choose the target category were omitted from analysis, as in all past experiments on this topic (e.g., [4], [5], [8], [16]).

### Results

As just described, when subjects did not identify the target as the most likely category, their induction score was omitted. In two stories, this resulted in fewer than ten valid responses across the two versions, so those stories were excluded from analysis. (We will discuss why subjects may not have chosen the target categories in detail in Experiment 2.) In the remaining stories, the target category was selected 69% and 72% of the time in the neutral and dangerous versions.

Our hypothesis was that when the alternative category was dangerous, subjects would take it into account when making an induction. Therefore, our analysis focused on two independent comparisons—whether the alternative category would influence inductions when it was a) dangerous and b) neutral. We first present the analysis of the corrected scores (Table 3, bottom). Consistent with our predictions, congruent inductions were rated as 10.4% higher (*SD* = 14.5) than incongruent inductions for the dangerous congruent question, *t*(30) = 4.0, *p*<.01, but not reliably higher for the neutral congruent question (*M* = 2.9, *SD* = 21.8), *t*(30) = 0.74. Because the latter finding is a null result, we carried out at 2×2 ANOVA with factors question type (congruent to the neutral or dangerous alternative) and alternative category mentioned in the story (neutral or dangerous) to see if the effects were different for the two types of questions. Indeed, there was a significant interaction between story and question type, *F*(1, 30) = 10.15, *p*<.01. There was also a main effect of question type, with the rating for dangerous questions (*M* = 7.1) significantly greater than the rating for neutral questions (*M* = 1.7), *F*(1, 30) = 4.90, *p*<.05. This main effect is not readily interpretable, as it reflects item differences. The difference between the neutral and dangerous stories was not significant *F*(1, 30) = 2.1, *p*>.10.

**Table 3:** Mean Probability Ratings from Experiment 1(Raw and Corrected Scores).

	Story Type		
Question Type	Neutral Alternative	Dangerous Alternative	Congruent minus Incongruent Induction
**Raw Scores**			
Neutral Congruent	**51.6**	49.4	2.1
Dangerous Congruent	23.4	**32.3**	8.9
**Corrected Scores**			
Neutral Congruent	**3.1**	0.2	2.9
Dangerous Congruent	1.9	**12.3**	10.4

*Note.* Corrected scores are feature prediction probability ratings minus target category ratings from pretesting. Congruent inductions are bolded.

Results for the raw data were similar, though the differences were slightly smaller. As shown in Table 3 (top), there was a difference between congruent and incongruent inductions when the question was congruent to the dangerous alternative (*M* = 8.9, *SD* = 17.6), *t*(30) = 2.82, *p*<.01, but no difference between congruent and incongruent inductions when the alternative was neutral (*M* = 2.1, *SD* = 27.1), *t*(30) = .44. In the 2×2 ANOVA, the interaction was reliable, *F*(1, 30) = 7.51, *p*<.01, along with higher ratings for neutral questions, *F*(1, 30) = 108.94, *p*<.001. Again, the latter result is not readily interpretable, as it reflects content differences among questions (e.g., perhaps people think fever is generally more likely than undergoing surgery). The difference scores that are our main interest keep the questions constant.

### Discussion

This experiment investigated whether people use single or multiple categories in category-based inductions when category membership was uncertain. When a target (most likely) category and an alternative (less likely) category were mentioned in a story, subjects incorporated both categories into their predictions when the alternative category was dangerous but not when it was neutral. The latter result replicates many past findings that people do not attend to alternative categories with neutral alternatives, e.g., [6], [7], or [8] (naive subjects). The new finding is that people did incorporate information about a dangerous alternative category such as bats, cancer, or kidnappers into their predictions.

## Experiments 2A and 2B

Experiment 1 found that people attend to a dangerous alternative even when they say that they do not believe it is the most likely category. We consider two related interpretations of this effect. One is that the dangerous alternative intrudes into working memory, thereby influencing the prediction. Normally, people are biased to focus only on one hypothetical alternative at a time [9], but the dangerous category draws attention to itself and so is incorporated into the prediction.

Alternatively, the presence of a dangerous category may make people more vigilant as a whole. (We thank Aaron Hoffman for raising this possibility.) Given that cancer or bats have been mentioned, people could simply become more attentive to *any* possibility, as part of a protective mechanism. As an analogy, if you heard a strange noise in your house at night, you might also then become more able to detect other deviations from the expected, such as an unusual smell or light. On this account, the mention of cancer or bats did not attract attention just to themselves but to all mentioned categories, via a vigilance mechanism.

Experiment 2 distinguished these two possibilities by making the *target* category dangerous. If Brian's throat pain is most likely due to cancer but perhaps is an infection, then the vigilance notion suggests that all of the categories will receive attention. Even though cancer might seem likely to draw all the attention to itself, it could actually make subjects more sensitive to the cold or infection possibilities by making them consider all possibilities more carefully. In contrast, the idea that dangerous categories intrude on working memory would suggest that a dangerous target would make people ignore the alternative categories.

In Experiment 2A, we made a simple change to Experiment 1, namely making the dangerous category the target, and then using the other two categories as alternatives. The story remained otherwise identical, allowing very close comparison to Experiment 1. (New features had to be derived for the former target/now alternative category, as described below.) However, this experiment largely failed, because the target category was not selected as most likely most of the time. That is, although the doctor told Brian that he probably had throat cancer, but there was a small chance he had a cold/infection, many subjects later said that Brian's throat pain was probably just a cold or infection (in Question 5). Among other problems, this meant that we did not have sufficient data to make the comparisons of alternative categories in many stories, because all such responses are omitted from the data analysis.

In Experiment 2B, we made a number of changes to our paradigm, outlined in the Method, to increase the number of valid responses. We address the unexpected rejection of the target category in the Discussion.

### Methods

There were 20 subjects in Experiment 2A and 24 in 2B. As explained above, Experiment 2A simply switched the categories mentioned as most and less likely, with minimal other changes. New features had to be constructed for some of the former target categories, to make them distinct from the other alternative category (e.g., a feature for a throat infection that would not be found in a cold). These were pre-tested as before. On average, the properties were rated 53% more likely for their congruent than for their incongruent alternative category (*SD* = 19%), which was similar to the ratings in Experiment 1.

Given the problems with 2A, we made more changes to the stories of 2B in order to increase their believability. One concern was that all the experimental stories had a dangerous outcome as the most likely. Perhaps this began to seem unreasonable. Therefore, we reduced the set of items to include only eight stories that had higher rates of target category selection in 2A, and we added four filler scenarios with no dangerous components. Next, we revised the stories so that the characters appeared to take the dangerous category seriously. Perhaps the fact that the original stories didn't remark on the terrible outcomes made them less believable. Now, after telling Brian the bad news about his throat, the doctor expressed concern and suggested an appointment to discuss the outcome of a biopsy. Thus, the characters now responded as if they believed the dangerous target category to be likely. The procedure was identical to that of Experiment 1.

### Results and Discussion

In Experiment 2A the target category was selected only 34% of the time. Experiment 2B was slightly better, at 47%. However, this figure was influenced by two stories where people chose the target categories very seldom, in spite of our changes. Therefore, we dropped them and focused on the six remaining stories, where the target categories were selected 56% of the time on average. Because Experiments 2A and 2B had the same categories and questions on these six shared stories, we combined them in analysis, for greater power (discarding the other, less successful stories). Altogether, selection of the target category was 53% for these shared stories, ranging from 34% to 89%.

The mean predictions for the congruent and incongruent features were 33.5 and 29.3, which were not reliably different, *t*(43) = 1.02. However, in the corrected scores (subtracting the probability of the target category for each feature), this difference became reliable: 10.2 vs. 4.1, *t*(43) = 2.35, *p*<.01. (Since all alternatives were neutral, there is no variable of category type, and hence no 2-way ANOVA in Experiment 2.) Although the difference is not large (only 6%), five out of six stories showed a higher probability rating for the congruent than the incongruent questions (for both raw and corrected scores). Thus, using a dangerous target category raised people's attention to all the mentioned categories.

Why were the results stronger in the corrected scores? This is primarily due to the missing data. If every subject had contributed data from every story, the correction would have had no effect, because it would have amounted to subtracting a constant from everyone's score. However, when different subjects contributed data from different stories, the inherent differences among those stories (and their questions) created differences between the subjects' means unrelated to the independent variables. By subtracting the target probability from each score, we accounted for this variability and hence lowered the amount of noise in the data.

The results support the vigilance account of Experiment 1's results. If the dangerous category had intruded into working memory, then that should have made people ignore the neutral alternative (as they did in Experiment 1). The fact that the alternative influenced induction shows that the dangerous target did not dominate attention.

The avoidance of the target category in Experiment 2A was unexpected. We have found in past research that subjects do not always agree with the stated most likely category, which is not surprising. When reading fiction, readers often disagree with the stated perceptions or conclusions of characters. Given that the stories express uncertainty about the object, some disagreement is to be expected (and when stories don't express uncertainty, that disagreement decreases [6]). But a success rate of only 34% is far below what we have found before [6], [7].

Clearly, subjects did not take it for granted when a character said, “It is most likely X but possibly could be Y,” that the entity was most likely X. Rejection of the dangerous target was much greater than rejection of the neutral target (target selected 57% vs. 34% of the time in Experiments 1 and 2A with almost identical stories, including all scenarios). We can look back at target selection in Experiment 1 to see if dangerousness of the *alternative* influenced it, and it did. A blind coder examined each response to the final classification question in Experiment 1 for all 12 scenarios and decided whether the response was the target or a related category. (Our original coding required that the target category be clearly indicated, omitting some vague responses such as “a student” or “a fish,” when a more specific category had been indicated. However, those responses are relevant to the present analysis of whether subjects avoided the dangerous alternative and so were included.) When the alternative was neutral, people chose the target or a related category 59% of the time. This increased to 72% when the alternative was dangerous, *t*(30) = 2.72, *p*<.02. Thus, a dangerous alternative caused a slight increase in selecting the nondangerous target. Taking these results together, it seems clear that people prefer not to choose a dangerous category as the most likely one.

## General Discussion

### Multiple Use of Categories in Induction

This study investigated how people make predictions about items whose category membership is uncertain. Several studies have shown that people often use only one category to make inductions without considering less likely alternative categories, even though they are not certain the object belongs to that category [4], [6], [7]. That finding was replicated in Experiment 1, with unthreatening target and alternative categories. But when the alternative category was dangerous, people used it in their predictions even when they claimed that the nondangerous target was the most likely outcome. Thus, they used multiple categories, contrary to the singularity principle.

This finding is consistent with Hayes and Newell's [16] results, but our experiment used familiar, natural categories and did not overtly draw attention to the dangerous nature of the categories. Apparently, bats and cancer are attention-getting enough that one does not need to mention their dangerous properties for people to take them into account in induction. However, Hayes and Newell's finding is also important, because it suggests that when dangerous categories are not familiar (e.g., novel diseases), pointing out their negative features can influence people to attend to them when making inductions.

In sum, the short answer to the question that motivated these experiments is that people may be more likely to attend to multiple potential categories when one of them is dangerous. Under the assumption that using all possible categories will lead to more accurate predictions, this suggests that people may be reasoning more normatively under these conditions. Our result also found unexpected results that we address below. Because they were not the focus of the research, this discussion is necessarily more speculative.

### Rejection of Dangerous Target Categories

In Experiment 2, people used multiple categories when the target itself was dangerous. Our original prediction of this possibility was based on the notion of vigilance, that the dangerous option would raise people's attention to all possible categories. The literature on *automatic vigilance* argues that attention is drawn to negative stimuli without conscious control, as shown in perceptual detection and interference effects [12], [13]. A similar mechanism might be involved in induction with a less dangerous category, in which the dangerous category cannot be easily excluded from the induction process even though it is less likely. Just as a picture of a snake attracts your attention even when it is irrelevant to what you are doing [12], [13], the possibility of a snake in your bed (as in one of our stories) is difficult to dismiss from your thoughts even if it is unlikely.

However, it is not clear that the vigilance interpretation is consistent with the finding that subjects avoided the dangerous targets (and alternatives, in Experiment 1). If your doctor tells you that you most likely have cancer, would a vigilant response be to decide that it is most likely a cold? It seems likely that a different mechanism, perhaps in conjunction with vigilance, is at work. One possibility is *motivated reasoning*. When people encounter threatening information, they are likely to adduce arguments that defuse it [18], [19]. If given a likely diagnosis of cancer (or bats flying at you or a being met on a dark street by a gang member), your thoughts may also jump to more positive possibilities: Further testing will reveal that the throat pain is something else; the bat is just a confused swallow; the gang member is just a street person. Although this represents some degree of wishful thinking, it is also important to note that when subjects did agree that the dangerous category was more likely, they also attended to the alternative category, making more normative responses.

Thus, the cost or benefit of reasoning about a dangerous possibility depends on whether it is a less likely alternative—when it may be correctly considered—or the most likely possibility—when it may be incorrectly rejected. The present experiments were not designed to investigate the avoidance of likely dangerous categories, so these possibilities clearly require further investigation.

A final possibility is that the lower frequency of dangerous outcomes might make subjects choose them less. Perhaps they are simply paying attention to base rates. We believe that there is probably an effect of frequency, but it may not be a straightforward one. Although toddlers only very infrequently ingest narcotics, in a situation in which a toddler has gotten into a cupboard containing colorful narcotic pills, it is probably not good statistical reasoning to think, “Toddlers very seldom eat painkillers, so I'm not that worried.” Similarly, if a doctor says that you most likely have throat cancer, the probability that you have throat cancer is presumably much higher than that of the average patient. The likelihood of a dangerous outcome has to be considered relative to the specific situation, and not simply in terms of overall base rates. It seems very possible that people may reason, “Throat cancer is very rare, so Brian probably doesn't have it,” but it is unclear whether frequency itself is the real factor or is an excuse seized on in a search for reasons to reject the target category.

### Limitations and Future Directions

The nature of this paradigm only allows us to make group comparisons. Some subjects received bats as an alternative category and others received robins. We then compared the inductions of those two groups. An individual subject's response cannot be identified as using single or multiple categories (see [5], for a technique for doing this with artificial categories). However, it is possible that our effects are being carried by individuals who happen to be more sensitive to some of these categories. The person who *really* doesn't like bats might be giving a large probability to the bat-congruent property, and other subjects aren't. In contrast, some of those subjects might be giving high probabilities to the cancer-congruent prediction. The effect sizes (especially in Experiment 2) seem smaller than would be expected by consistent attention to the alternative category, making us suspect that they are an average of attentive and nonattentive responses.

We should also note that not only the categories but also their related features were typically undesirable. For example, throat cancer (category) and surgery (predicted feature) are both unwanted. It may be that both must be dangerous to some degree for the effect to hold. Separating the two is difficult, because strongly undesired categories tend to have undesired properties, and a neutral category would hardly be neutral if it had a dangerous property (e.g., poisonous toast).

The results of the current study provide further evidence that people can use multiple categories to make category-based inductions in certain contexts [7], [8]. Heuristics and other shortcuts of the cognitive miser often lead people to focus on a single most likely category, but the presence of dangerous information can promote the consideration of alternative categories. However, this may not be true for every situation involving dangerous categories. For example, if several alternatives were dangerous (as in diagnosis of a serious medical condition), would people incorporate them all into their decision-making? Further research should investigate such situations to gain a greater understanding of the effects of dangerous possibilities on how people make inductions when category membership is uncertain.

Further research should also investigate these issues in other populations. Our subjects were students at an American university, and it is possible that responses to dangerous categories vary with age or education. Cultural differences may also be expected, as people in different cultures may respond differently to the same emotions [20]. Furthermore, one proposed difference across cultures is in how their members deal with uncertainty and sufficiency. According to Nisbett et al. [21], Western cultures take an either-or approach to truth, whereas East Asian cultures are more accepting of multiple truths and multiple causes as explanations. That seems very related to the issues of single vs. multiple category use underlying our experiments. Thus, our paradigm provides one potential way to investigate cultural differences in how people respond to such situations.
